# Using telehealth to support antimicrobial stewardship at four rural VA medical centers: Interim analysis

**DOI:** 10.1017/ash.2023.387

**Published:** 2023-09-29

**Authors:** Alexandria Nguyen, Mayyadah Alabdely, Taissa Bej, Tola Ewers, Tammy Walkner Amanda Vivo, Christopher Crnich, Daniel Livorsi, Rabeeya Sabzwari, Geneva Wilson, Brigid Wilson, Corinne Kowal, Oteshia Hicks, Charlesnika Evans, Robin Jump

## Abstract

**Background:** Healthcare settings without access to infectious diseases experts may struggle to implement effective antibiotic stewardship programs. We previously described a successful pilot project using the Veterans Affairs (VA) telehealth system to form a Videoconference Antimicrobial Stewardship Team (VAST) that connected multidisciplinary teams from rural VA medical centers (VAMCs) with infectious diseases experts at geographically distant locations. VASTs discussed patients from the rural VAMC, with the overarching goal of supporting antibiotic stewardship. This project is currently ongoing. Here, we describe preliminary outcomes describing the cases discussed, recommendations made, and acceptance of those recommendations among 4 VASTs. **Methods:** Cases discussed at any of the 4 participating intervention sites were independently reviewed by study staff, noting the infectious disease diagnoses, recommendations made by infectious diseases experts and, when applicable, acceptance of those recommendations at the rural VAMC within 1 week. Discrepancies between independent reviewers were discussed and, when consensus could not be reached, discrepancies were discussed with an infectious diseases clinician. **Results:** The VASTs serving 4 different rural VAMCs discussed 96 cases involving 92 patients. Overall, infection of the respiratory tract was the most common syndrome discussed by VASTs (Fig. 1). The most common specific diagnoses among discussed cases were cellulitis (n = 11), acute cystitis (n = 11), wounds (n = 11), and osteomyelitis (n = 10). Of 172 recommendations, 41 (24%) related to diagnostic imaging or laboratory results and 38 (22%) were to change the antibiotic agent, dose, or duration (Fig. 2). Of the 151 recommendations that could be assessed via chart review, 122 (81%) were accepted within 1 week. **Conclusions:** These findings indicate successful implementation of telehealth to connect clinicians at rural VAMCs with an offsite infectious diseases expert. The cases represented an array of common infectious syndromes. The most frequent recommendations pertained to getting additional diagnostic information and to adjusting, but not stopping, antibiotic therapy. These results suggest that many of the cases discussed warrant antibiotics and that VASTs may use the results of diagnostic studies to tailor that therapy. The high rate of acceptance suggests that the VASTs are affecting patient care. Future work will describe VAST implementation at 4 additional VAMCs, and we will assess whether using telehealth to disseminate infectious diseases expertise to rural VAMCs supports changes in antibiotic use that align with principles of antimicrobial stewardship.

**Figure:**
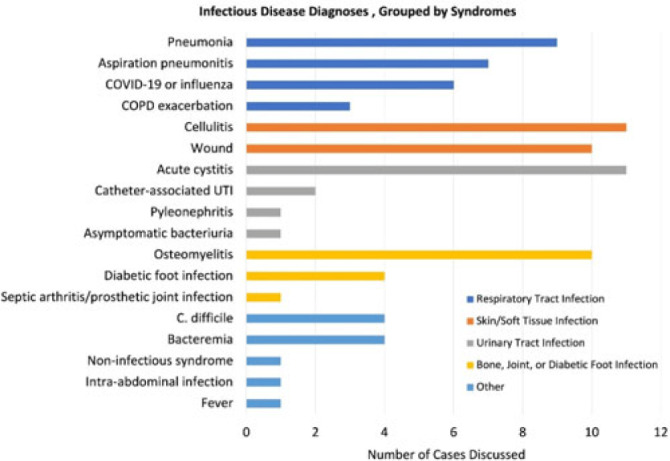

**Figure:**
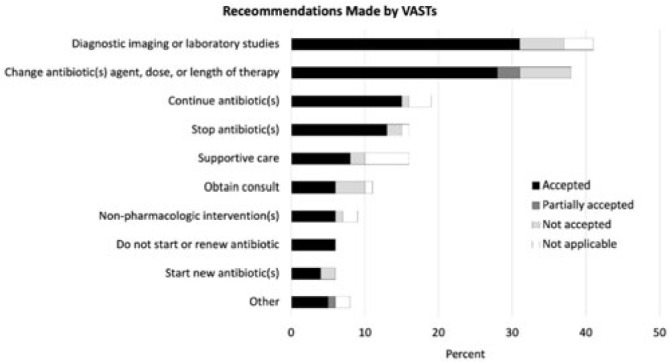

**Disclosures:** None

